# Effect of Face Blurring on Human Pose Estimation: Ensuring Subject Privacy for Medical and Occupational Health Applications

**DOI:** 10.3390/s22239376

**Published:** 2022-12-01

**Authors:** Jindong Jiang, Wafa Skalli, Ali Siadat, Laurent Gajny

**Affiliations:** 1 Institut de Biomecanique Humaine Georges Charpak, Arts et Metiers Institute of Technology, 75013 Paris, France; 2 Laboratoire de Conception Fabrication Commande, Arts et Metiers Institute of Technology, 57070 Metz, France

**Keywords:** face blurring, deep learning, human pose estimation, kinematic analysis

## Abstract

The face blurring of images plays a key role in protecting privacy. However, in computer vision, especially for the human pose estimation task, machine-learning models are currently trained, validated, and tested on original datasets without face blurring. Additionally, the accuracy of human pose estimation is of great importance for kinematic analysis. This analysis is relevant in areas such as occupational safety and clinical gait analysis where privacy is crucial. Therefore, in this study, we explore the impact of face blurring on human pose estimation and the subsequent kinematic analysis. Firstly, we blurred the subjects’ heads in the image dataset. Then we trained our neural networks using the face-blurred and the original unblurred dataset. Subsequently, the performances of the different models, in terms of landmark localization and joint angles, were estimated on blurred and unblurred testing data. Finally, we examined the statistical significance of the effect of face blurring on the kinematic analysis along with the strength of the effect. Our results reveal that the strength of the effect of face blurring was low and within acceptable limits (<1°). We have thus shown that for human pose estimation, face blurring guarantees subject privacy while not degrading the prediction performance of a deep learning model.

## 1. Introduction

Human pose estimation is a highly important task in the field of computer vision. The focus of human pose estimation is the calculation of human body keypoint coordinates based on images. Combined with kinematic analysis, it has the potential for many applications in different fields, e.g., ergonomics [1,2,3,4] or orthopedics [5,6]. In vision-based human pose estimation tasks, the datasets used for training and testing models often consist of images where the human face is clearly visible. In applications, this fact raises a significant privacy problem. For instance, in ergonomics, vision-based human pose estimation can help workers prevent musculoskeletal disorders [4,7,8,9,10]. However, installing cameras in factories to capture workers’ motion leads to significant privacy concerns and workers might legitimately reject this tool. As a result, addressing the privacy issues of computer vision datasets is an essential task. Fortunately, simple face blurring could solve most of these privacy concerns. However, the effect of face blurring on human pose estimation and subsequent kinematic analysis is unclear.

Preservation of subject privacy in datasets for computer vision tasks is an emerging research topic. So far, few studies have investigated approaches, such as face blurring, to preserve subject privacy with a minimal impact on the performance of deep learning models [11,12,13,14,15,16,17,18,19,20,21]. Specifically, in [16], the authors proposed a large-scale face detection and blurring algorithm but did not quantify the impact of this anonymization on any computer vision task. In another work [14], the possibility of using adversarial training methods was explored to remove privacy-sensitive features from faces while minimizing the impact on action recognition. However, the statistical significance of this impact was not analyzed. In [17], the authors quantified a performance reduction for video action classification due to face blurring, and also proposed a generalized distillation algorithm to mitigate this effect. Similarly, a self-supervised framework was proposed in [13] for action recognition to eliminate privacy information from videos with no need for privacy labels. In [11], research has been carried out using a large-scale dataset to examine more comprehensively the effect of face blurring on different computer vision tasks. The authors first annotated and blurred human faces in ImageNet [22]. Afterward, they benchmarked several neural network models using the face-blurred dataset to examine the effect of face blurring on the recognition task. Finally, with the models pre-trained on the original/face-blurred dataset, they studied the feature transferability of these models on “object recognition, scene recognition, object detection, and face attribute classification”. The results of the experiment suggested that, in the vision tasks above, face blurring did not cause a significant loss of accuracy. In close connection with our study, a facial swapping technique has been applied using videos of patients with Parkinson’s disease and 2D human pose estimation was performed in [15]. The authors concluded that facial swapping keeps the 2D keypoints almost invariant, but this study was limited to only two subjects.

Therefore, previous works have focused on the task of classification, action recognition, or 2D human pose estimation in videos. Thus far, the effect of face blurring on 3D human pose estimation and, more importantly, subsequent kinematic analysis has not yet been investigated on a consistent cohort. Considering their importance in biomechanical and ergonomic domains, we study the statistical significance as well as the strength of the effect of face blurring on 3D human pose estimation and kinematic analysis in this paper. Our contribution consists of three main parts. First, to the best of our knowledge, this study is the first one focusing on the effect of face blurring on multi-view 3D human pose estimation. Second, based on the 3D keypoint coordinates obtained from the human pose estimation, we calculated joint kinematics and analyzed the eventual impact of face blurring. Third, both statistical significance and strength were calculated to more comprehensively evaluate the effect of face blurring on the performance of a deep learning model.

## 2. Materials and Methods

**Figure 1:**
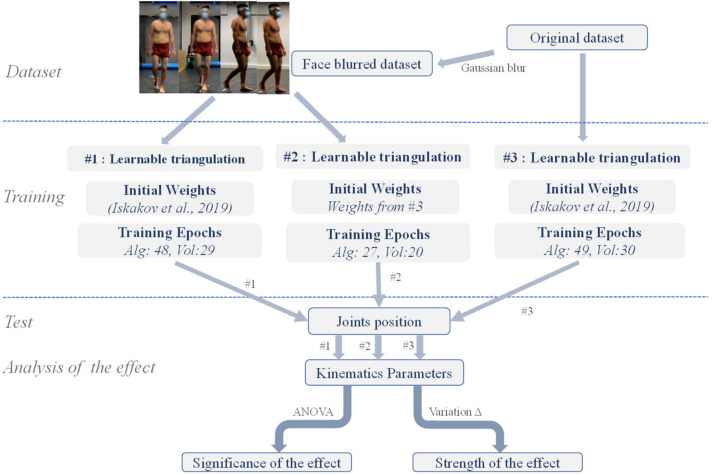
Flowchart of this study.

In this study, we first performed subject face blurring on an image dataset acquired in our previous gait study. Then, using different training strategies, we obtained distinct deep learning models and tested the performance of each model on the face blurring or the original dataset, thus examining the effect of face blurring. Figure 1 outlines the research flow of the work in this paper.

### 2.1. Dataset

**Figure 2:**
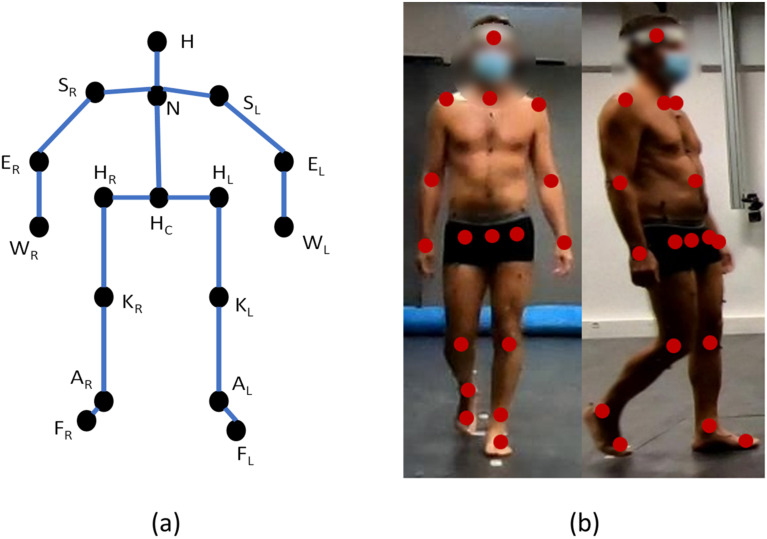
(**a**) The human model defined in the ENSAM dataset. (**b**) The 17 keypoints projected on two camera views.

The dataset used in this research was a multi-view human gait dataset, namely, the ENSAM dataset collected in a previous study [5]. The dataset contained a total of 43 subjects (19 females and 24 males; age range: 6–44 years; weight: 56.0 ± 20.7 kg; height: 159.2 ± 21.5 cm) which were split into a training set of 27 subjects (14 females and 13 males; age range: 8–41 years; weight: 54.0 ± 20.2 kg; height: 157.7 ± 21.8 cm) and a test set of 16 subjects (5 females and 11 males; age range: 6–44 years; weight: 59.6 ± 21.8 kg; height: 161.8 ± 21.3 cm). In the training set, 14 subjects were asymptomatic adults (≥18 years), one adult had scoliosis, and 12 minors (<18 years) suffered from X-linked hypophosphatemia (XLH) disease. While in the test set, 8 adults were asymptomatic, one adult had spondylolisthesis, and 7 children had XLH [5,6]. The dataset comprised a total of 120,293 frames, each containing four images from four calibrated and synchronized cameras (GoPro Hero 7 Black). The 3D positions of 51 markers attached to the body of subjects were captured by a marker-based motion capture system (VICON system, Oxford Metrics, Oxford, UK). The camera parameters of four cameras and biplanar radiographs acquired by the X-ray system (EOS system, EOS imaging, Paris, France) were also collected. With the help of the markers’ 3D positions acquired from the Vicon system along with the 3D reconstructions of lower limbs from bi-planar radiographs [23], the 3D coordinates of 17 keypoints were annotated on the human body. As shown in Figure 2, the keypoints were, namely, head (H), neck (N), shoulders ($S_{R}$, $S_{L}$), elbows ($E_{R}$, $E_{L}$), wrists ($W_{R}$, $W_{L}$), pelvis ($H_{C}$), hips ($H_{R}$,$\;H_{L}$ ), knees ($K_{R}$, $K_{L}$), ankles ($A_{R}$, $A_{L}$), and feet ($F_{R}$, $F_{L}$). 

**Figure 3:**
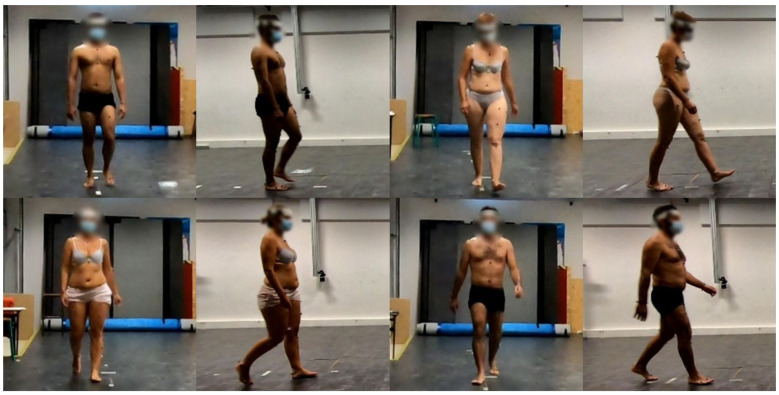
Example images of the ENSAM Pose dataset after face blurring.

With the camera parameters collected in the ENSAM dataset, the reference annotations of the head and the neck were projected onto the corresponding images. A circle covering the subject’s face was drawn based on these projections in the images. The pixels inside the circle were blurred using Gaussian blur. A Gaussian kernel size of 25 × 25 was carefully selected with which the faces of different sizes could all be blurred properly. The standard deviation of the kernel was set to 4.1 and determined automatically using OpenCV [24]. Using this method, face blurring of the images of all 43 subjects in the dataset was performed. Figure 3 shows some example images after face blurring.

### 2.2. Experiment Setup

As in [5], the 3D human pose estimation algorithm applied in this paper was the learnable triangulation algorithm proposed by [25]. The algorithm consists of two main parts, namely, 2D and 3D human pose estimation. The 2D human pose estimation was performed for each camera view, and subsequently, the information from all views was fused to derive the 3D coordinates of keypoints of the human body using a trainable triangulation approach. Two approaches were proposed in the article, i.e., the algebraic and the volumetric triangulation, where the latter required the pelvis position to be estimated by the former. 

Three training experiments were conducted, namely, #1, #2, and #3. The model training setup is summarized in Figure 1. In the experiments, original images or face-blurred images were utilized as the training set, with initial weights of the network being those provided in [25] or those acquired from the training of experiment #3. It is worth mentioning that the weights provided in [25] were obtained by training the network on the Human 3.6M dataset [26]. Regarding the number of training epochs, in experiments #1 and #3, the algebraic and the volumetric module were trained for 50 and 30 epochs, respectively, whereas in experiment #2, the 2 modules were finetuned for 30 and 20 epochs, respectively. At the end of each training epoch, we recorded the current model performance and the network weights. After the model training reached the number of epochs as described above, in each experiment, the epochs with the minimum error on the test set were then selected for the subsequent inference. The number of training epochs for the selected model is listed in Figure 1. Three models were obtained from the experiments, where models #1 and #2 were the experimental models, and model #3 was the control model. The training was performed using the Adam optimizer, with learning rates of 10^−5^ and 10^−4^ for the algebraic and the volumetric network, respectively. The training and evaluation of the neural network during the experiment was effectuated on a Linux server under Ubuntu 20.04.1 LTS 64 bits. The machine consisted of an AMD Ryzen 9 3900X 12-core processor and 125 GB RAM. It was equipped with 2 Nvidia TITAN RTX GPUs with 24 GB of RAM, one of which was employed in this study. 

For the three models obtained in the experiments, their performances for human pose estimation and the subsequent kinematic analysis were analyzed on the original or the face-blurred test set. 

### 2.3. Joint Angles Calculation

**Figure 4:**
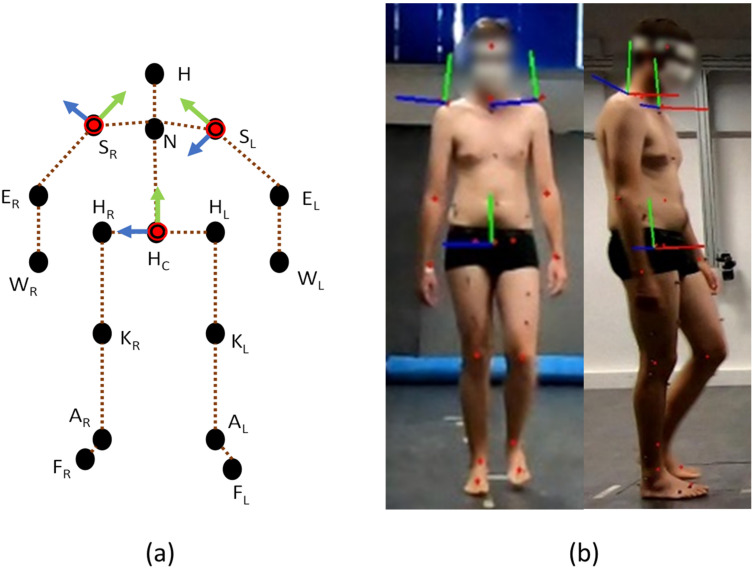
(**a**) Schematic diagram of the coordinate systems of shoulders and trunk. (**b**) The 17 keypoints and the coordinate systems of shoulders and trunk projected on two camera views, where the red, green, and blue axis represent the x, y, and z-axis, respectively, with a length of 20 cm in the world global coordinate system.

The human pose was defined by the 3D coordinates of the 17 keypoints in our human model. Using the 17 coordinates, joint angles were computed for the lower and upper extremities. For the joint angles of the lower limbs, we followed the calculation method proposed in [6]. For the upper limbs, the approach employed in [27] was modified to fit the human model defined in this study, and we applied it to establish the local coordinate system of the segment and calculate the corresponding joint angles (see Figure 4). 

The coordinate system of the trunk was defined as follows: The Y-axis is the vector $\vec{H_{C}N}$. The X-axis was perpendicular to the Y-axis and $\vec{H_{R}H_{L}}$. Then, the Z-axis was calculated from the X-axis and Y-axis according to the right-hand rule. The coordinate system origin was placed at $H_{C}$, and all axes were normalized to unit vectors.

The method employed in [27] was adopted for the computation of neck flexion, neck side bend, and elbow flexion, as well as the definition of shoulder coordinate systems. The rotation matrices of the shoulder coordinate systems relative to the trunk coordinate system were calculated. As suggested by [28], ZXY decomposition was performed to calculate the joint angles of the shoulder. 

### 2.4. Evaluation

The evaluation metric MPJPE (Mean Per Joint Position Error) was chosen in this paper to examine the performance of different models by comparing the joint positions estimated via the neural networks against the references acquired from the marker-based motion capture system. MPJPE was computed as follows [29]:

(1) $$\mathrm{MPJPE}=\frac{\sum_{i=1}^{N}\left|\left|J_{k}-\hat{J_{k}}\right|\right|}{N}$$

where $\hat{J_{k}}$ and $J_{k}$ denote respectively the estimated and the reference 3D position of the keypoint k. N is the total number of keypoints. For each keypoint, the effect of face blurring was analyzed by calculating the maximum variation Δ due to the face blurring, i.e., Δ*_j_* = *|μ_max_ − μ_min_|_j_*, where the *μ_max_* and *μ_min_* were the maximum and minimum values among all the 3 inference results estimated with different models on joint *j*. 

To analyze the effect of face blurring on kinematic calculations, we performed statistical tests on joint angles differences (with the reference system) calculated using different data sets based on different models.

Since the accuracies of human pose estimation on the original and the face-blurred test set are subject-by-subject matched, a one-way repeated ANOVA test was used to examine the effect of face blurring. In this test, we had one within-subject factor, namely, experiment setup, which had three levels, i.e., experiment #1, #2, and #3. The root mean square error (RMSE) of the joint angle of each subject was used as the dependent variable. We assumed that our data were consistent with the assumption of sphericity since different joint angle calculations did not affect each other. 

Finally, to further evaluate the strength of these effects, we quantitatively investigated the variations in joint angle estimation using different models. As a measure of the strength of the effect, the maximum variation Δ of angle estimation due to face blurring was defined for each joint *j*, i.e.,

(2) $$\Delta_{j}\left(RMSE\right)={\left|RMSE_{max}-RMSE_{min}\right|}_{j}$$

(3) $$\Delta_{j}\left(SD\right)={\left|SD_{max}-SD_{min}\right|}_{j}$$

where $RMSE_{max}$ and $RMSE_{min}$ were respectively the maximum and minimum values among all the inference results estimated with different models on joint angle *j*, with $SD_{max}$ and $SD_{min}$ being those of the standard deviations of the differences.

## 3. Results

### 3.1. Evaluation of Joint Position

Joint localization performances from the above experiments are shown in Table 1, where the mean and standard deviation of the prediction errors for each joint are reported. Notably, the differences of 3D keypoint coordinates in the lower extremities (ankles, knees, hips, and pelvis) were lower than those in the upper extremities (neck, head, wrists, elbows, and shoulders). The range of variation in the average difference of all joints was less than 1 mm (see MPJPE column in Table 1). Experiment #3 (on original images) and experiment #1 (on face-blurred images) achieved comparable performance (MPJPE = 13.3 mm vs. 13.0 mm). On the other hand, the performance of model #2, which was first trained on the original images and then fine-tuned on the face-blurred images, did not show any improvement (MPJPE = 13.4 mm).

The keypoints with the highest variations were the head and neck, with a Δ of 2.7 mm and 1.0 mm, respectively. For the head, the best- and worst-performing settings were #1 (*μ* = 11.1) and #3 (*μ* = 13.8), respectively. Unlike the head, #1 (*μ* = 11.0) and #3 (*μ* = 10.0) were the worst- and best-performing settings for the neck, respectively. 

### 3.2. Kinematics Analysis 

#### 3.2.1. Statistical Significance of the Effect of Face Blurring

**Figure 5:**
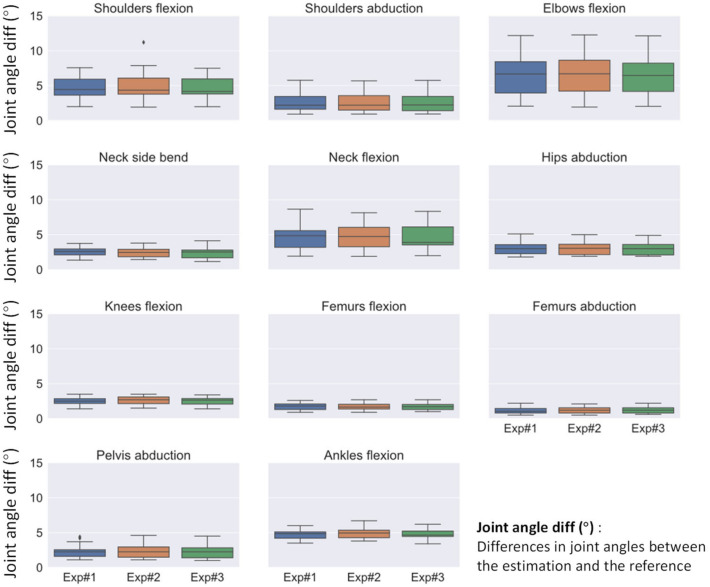
Box plots of the distribution of joint angle differences (RMSEs of the frames for each subject in the testset).

Figure 5 presents an overview of joint angle estimation differences (the RMSE values being calculated for each subject in the test set). For all the models, knee flexion, femur abduction, and femur flexion were the angles estimated as having the smallest differences to the reference. On the other hand, elbow flexion, neck flexion, and shoulder flexion were the angles with the largest differences. Nevertheless, neither for the upper limbs nor lower limbs, no variation larger than 5° was observed in the differences of joint angles estimated with different models. The significance of the effects of face blurring on the angle estimation differences was then revealed by statistical tests.

The statistical test results shown in Table 2 were the *p*-values of one-way repeated measures ANOVA tests for each joint angle. In the ANOVA test, our null hypothesis was that there was no variation in the mean value of the angle calculation RMSE in different experiments. Therefore, when the *p*-value was lower than 0.05, we rejected the null hypothesis whereby the effect of face blurring on corresponding joint angle calculations was considered statistically significant. From the results, no statistically significant variation was found on all joint angle calculations except one. The elbow flexion was affected with statistical significance (*p*-value = 0.040) by the different experiment setups.

#### 3.2.2. Strength of the Effect of Face Blurring

We have presented the statistical significance of the effect of face blurring on the joint angle calculation using ANOVA tests. To further investigate the strength of the effect of face blurring, variations of joint angle computation were quantified.

Table 3 provides the root mean square values and standard deviations of the errors of joint angles estimated on the face-blurred and original test sets via the models obtained from the experiments. Most RMSE values did not exceed 5°. Only shoulder flexion (RMSE: 5.5, SD: 5.3), neck flexion (RMSE: 5.2, SD: 5.2), and the elbow flexion (RMSE: 7.4, SD: 6.7) were above this limit, and the maximum values came from model #2.

The maximum variations Δ of joint angles are also presented in Table 3. Overall, the maximum variation Δ of the RMSE (+SD of all the frames) was smaller than 1° for all joint angles. It is worth pointing out that the largest variation was observed for shoulder flexion, which was 0.6 (0.6).

## 4. Discussion

In this study, we aimed to assess the significance of the effect of face blurring on landmark localization performances and the effect on the subsequent kinematic analysis. To that end, a comparison between a control model (#3: trained and evaluated on unblurred images) and models trained or finetuned (#1 and #2) and evaluated on blurred images was led.

Concerning keypoint localization, regardless of the different experiments, the errors in the upper extremities (neck, head, wrists, elbows, and shoulders) were larger than those in the lower extremities (ankles, knees, hips, and pelvis). One possible reason was that the annotations in the training set of lower limb keypoints were refined by the 3D reconstructions from the biplanar radiographs. Another important reason is that the arm activities during movement show more variation between different subjects, so the algorithm is less robust when predicting the keypoints’ positions.

Regarding the average performance, the MPJPE of experiment #1 was comparable to that of experiment #3. For experiment #2, the MPJPE decreased marginally, indicating that we can train the model directly on the face-blurred images without pre-training on the original images. As expected, the most impacted keypoints by face blurring were the head and neck. Surprisingly, the head localization showed lower differences in model #1 than the control model #3. On the other hand, the other keypoints were impacted almost negligibly (maximum average variation was 0.7 mm).

In addition to the experiments presented in this paper, we also evaluated the performance of the three models for all possible combinations with both blurred/original test sets. As expected, we found that when the training data were of different types than the test data, keypoint localization performance slightly decreased.

One-way repeated measures ANOVA tests revealed that elbow flexion was statistically significantly affected, although for all other joint angle calculations we did not observe a statistically significant effect of different experimental settings on kinematic calculations. However, whether the strength of these effects was within acceptable limits needed to be analyzed quantitatively. As stated in [30], a joint angle estimation is “regarded as reasonable” if the difference is less than 5°. Therefore, in this paper, 5° was adopted as an acceptable difference. In other words, the joint angle estimation was considered acceptable when the difference between the angle estimated with the marker-less and the marker-based motion capture systems was less than 5°. 

We have demonstrated in Table 3 that the maximum variation of the RMSE of joint angles was negligible (0.6°), implying that there was little difference between the central values of joint angle confidence intervals. Meanwhile, RMSE ± 1.96×SD is the 95% confidence interval of the joint angle estimate; therefore, we can consider that face blurring does not have a strong impact on the kinematic analysis of one joint angle if the maximum variation Δ of SD on this angle is less than 5°/(1.96 × 2), which is 1.27°. In our results, only slight variations Δ of SD (less than 1°) were observed for most of the joint angles. Even so, closer inspection of the results showed that in accord with our observations in the previous analyses, the most affected angle was the shoulder flexion, with variations of 0.6°. Even for the elbow flexion, the calculation of which was deemed to be impacted with a high statistical significance, the maximum variations Δ of SD was also less than 1°, demonstrating that the impact of face blurring on the calculation of this angle is still acceptable.

There are also several limitations in this present study. The dataset used in the research was a gait dataset, and other types of movements were not investigated. Most subjects in our dataset were masked because of the context of the COVID-19 pandemic. Moreover, a single camera setup was investigated. It would be then interesting to examine the effect of face blurring on other datasets including other motions [2,26], unmasked subjects, and different camera setups. 

## 5. Conclusions

In this study, we present the first comprehensive investigation of the effect of face blurring on 3D human pose estimation. We have performed subject face blurring on an image dataset acquired in a previous gait study and investigated the impact of face blurring on human pose estimation and the subsequent kinematic analysis. Following this, we examined the statistical significance of the effects of face blurring on joint angle calculations with a further analysis of the strength of these effects. The results show that training the model on face-blurred images does not have a large impact on the performance of the model. The effects of face blurring are not found statistically significant on kinematic calculations for all joint angles except one (elbow flexion; however, this effect is relatively weak and acceptable). Moreover, we can train the neural network directly on face-blurred images without pre-training on the original images. Our findings indicate that it is feasible to utilize face-blurred image datasets for human pose estimation and effectively protect the privacy of subjects in training datasets without loss of performance in the subsequent kinematic analysis, thus facilitating data sharing that can accelerate convergence of clinical or ergonomic applications.

## Figures and Tables

**Table 1:** Inference results with the models trained on blurred/original images. *μ*: the mean value of joint position differences with the reference system (mm). σ: standard deviation (mm).

	Feet	Ank.^a^	Knees	Hips	Pelvis	Neck	Head	Wrists	Elbows	Sho.^s^	MPJPE
inference on face-blurred images, with model #1, trained on face-blurred images											
*μ*	10.1	7.2	11.0	15.8	12.7	11.0	11.1	14.7	15.2	21.7	13.0
2σ	13.6	9.9	11.2	15.0	11.0	11.3	11.9	23.2	19.9	24.1	/
inference on face-blurred images, with model #2, finetuned on face-blurred images											
*μ*	9.7	7.2	10.7	16.3	12.9	10.4	13.3	15.4	15.4	22.3	13.4
2σ	13.0	9.9	10.5	15.8	11.5	11.6	14.1	34.2	21.4	38.7	/
inference on the original images, with model #3, trained on the original images											
*μ*	9.8	7.1	11.0	16.4	13.1	10.0	13.8	15.0	15.4	21.8	13.3
2σ	13.1	10.2	12.2	17.0	11.7	11.4	14.3	27.7	24.4	36.0	/
variation between the maximum and minimum values of *μ*											
Δ	0.4	0.1	0.3	0.6	0.4	1.0	2.7	0.7	0.2	0.6	0.4

^a^ ankles, ^s^ shoulders.

**Table 2:** Results (*p*-value) of one-way repeated measures ANOVA tests for the effect of face blurring on kinematics calculation. Numbers with underscores suggest significant effects (*p*-value < 0.05).

Joint Angles	*p*-Value
shoulder flexion	0.259
shoulder abduction	0.338
elbow flexion	0.040
neck side bend	0.237
neck flexion	0.896
hip abduction	0.895
knee flexion	0.320
femur flexion	0.931
femur abduction	0.217
pelvis abduction	0.756
ankle flexion	0.106

**Table 3:** The RMSE (+SD of all the frames) of joint angles calculated with the joints’ position from the models trained on blurred/original images (°).

Joint Angles	Model #1 Trained on Blurred Images Inference on Blurred Images	Model #2 Finetuned on Blurred Images Inference on Blurred Images	Model #3 Trained on Original Images Inference on Original Images	Δ
shoulder flexion	4.9 (4.7)	5.5 (5.3)	4.9 (4.7)	0.6 (0.6)
shoulder abduction	2.9 (2.9)	3.1 (3.1)	3.1 (3.1)	0.2 (0.2)
elbow flexion	7.2 (6.5)	7.4 (6.7)	7.1 (6.4)	0.3 (0.3)
neck side-bend	2.7 (2.2)	2.6 (2.2)	2.6 (2.2)	0.1 (0.0)
neck flexion	5.1 (5.1)	5.2 (5.2)	5.2 (5.2)	0.1 (0.1)
hip abduction	3.2 (3.1)	3.2 (3.1)	3.2 (3.1)	0.0 (0.0)
knee flexion	2.6 (2.6)	2.6 (2.6)	2.6 (2.6)	0.0 (0.0)
femur flexion	1.8 (1.8)	1.8 (1.8)	1.8 (1.8)	0.0 (0.0)
femur abduction	1.2 (1.2)	1.3 (1.3)	1.3 (1.3)	0.1 (0.1)
pelvis abduction	2.6 (2.5)	2.6 (2.6)	2.6 (2.5)	0.0 (0.1)
ankle flexion	4.7 (4.6)	5.0 (4.9)	4.8 (4.7)	0.3 (0.3)

## Data Availability

Not applicable.
